# Frequent and strong antibody-mediated NK cell activation to HIV-1 Env in individuals with chronic HIV-1 infection

**DOI:** 10.1186/1742-4690-9-S2-P171

**Published:** 2012-09-13

**Authors:** C Thobakgale, L Fadda, K Lane, I Toth, F Pereyra, S Bazner, T Ndung'u, BD Walker, E Rosenberg, G Alter, M Carrington, T Allen, M Altfeld

**Affiliations:** 1 University of KwaZulu-Natal, Durban, South Africa; 2 Ragon Institute of MIT, MGH and Harvard, Boston, MA, USA; 3 Cancer and Inflammation Program, Laboratory of Experimental Immunology, Frederick, MD, USA

## Background

Natural killer (NK) cells are critical in viral control and NK cells that respond to HIV-1 peptides have been described. However, it is unclear how NK cells recognize HIV-1 antigen. We investigated NK cell responses to HIV-1 peptides during early and chronic HIV-1 clade B infection.

## Methods

NK cells responding to HIV-1 peptides were assessed by multiparameter flow cytometry using whole blood from 74 individuals with treated or untreated early or chronic HIV-1 infection. In addition, 15 HIV uninfected individuals were also studied.

## Results

No NK cell responses to HIV-1 peptides were detected in HIV-1 uninfected individuals. The HIV-1 NK cell specific responses to peptide were less frequent during the first year of infection but were of high magnitude and frequent in individuals with controlled or progressive infection (22% vs 79%; P<0.00001). The activation of NK cells to peptide pools required the presence of plasma IgG and the responding NK cells had a low CD16 expression and high CD57 expression. Furthermore, plasma derived from HIV-1 infected individuals was sufficient to trigger a response to HIV-1 Env peptide pool by NK cells from healthy donors suggesting the role of antibodies in mediating this activity.

## Conclusion

NK cell responses to specific antigens can be induced in HIV-1 infection. Large cohorts are needed to assess the consequences of these NK cells against HIV-1 control or protection from infection.

